# Impact of Electron‐Withdrawing Passivating Molecules on Perovskite Solar Cells

**DOI:** 10.1002/cssc.202501503

**Published:** 2025-10-09

**Authors:** Guangyue Yang, Kaiwen Dong, Lina Zhu, Yu Lei, Panyu Wang, Na Shi, Likai Zheng, Xiaoqing Jiang, Juan‐Ding Xiao

**Affiliations:** ^1^ College of Chemical Engineering Qingdao University of Science and Technology Qingdao 266042 China; ^2^ Laboratory of Photonics and Interfaces École polytechnique fédérale de Lausanne 1015 Lausanne Switzerland; ^3^ Institutes of Physical Science and Information Technology Anhui Graphene Carbon Fiber Materials Research Center, Anhui University Hefei Anhui 230601 P. R. China

**Keywords:** electron‐withdrawing materials, interface modification, perovskite solar cells

## Abstract

Interfacial instability—originating from defects, ion migration, and uncontrolled crystallization—remains a critical challenge for perovskite solar cells (PSCs). Electron‐withdrawing materials (EWMs) have emerged as promising candidates for interface engineering due to their distinctive coordination capability and favorable charge extraction properties. This review outlines the fundamental characteristics of EWMs and the underlying mechanisms by which they enhance photovoltaic performance and device stability. Recent advances in incorporating such materials across key interface layers are systematically summarized, offering mechanistic insights and design strategies toward the development of efficient and stable PSCs.

## Graphical abstract

The work reviews the effect of electron‐withdrawing groups on the photoelectric performance and stability of perovskite solar cells (PSCs), mainly focusing on the mechanism by which the group improves the performance and film quality of PSCs.

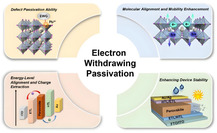

## 1 Introduction

In recent years, organic‐inorganic hybrid perovskite solar cells (PSCs) have garnered significant attention due to their high photovoltaic conversion efficiency (PCE), tunable optical bandgap, high dielectric constant, strong light absorption coefficient, and low crystallization barrier.^[^ ^1^, ^2^, ^3^, ^4^ ^]^ Over the past decade, the power conversion efficiency of single‐junction PSCs in small‐scale devices has reached over 27%, driven by rapid advancements in material processing techniques and device design optimizations. Grätzel et al.^[^ ^5^ ^]^ demonstrated that one‐step fabrication methods, combined with annealing of the absorber layer at 100 °C, yield PSCs with optimal efficiency. Jeon further revealed that chemical approaches to material crystallization, such as drop‐casting antisolvent during spin‐coating, enable the formation of high‐quality and dense thin films.^[^ ^6^ ^]^

**Figure 1:**
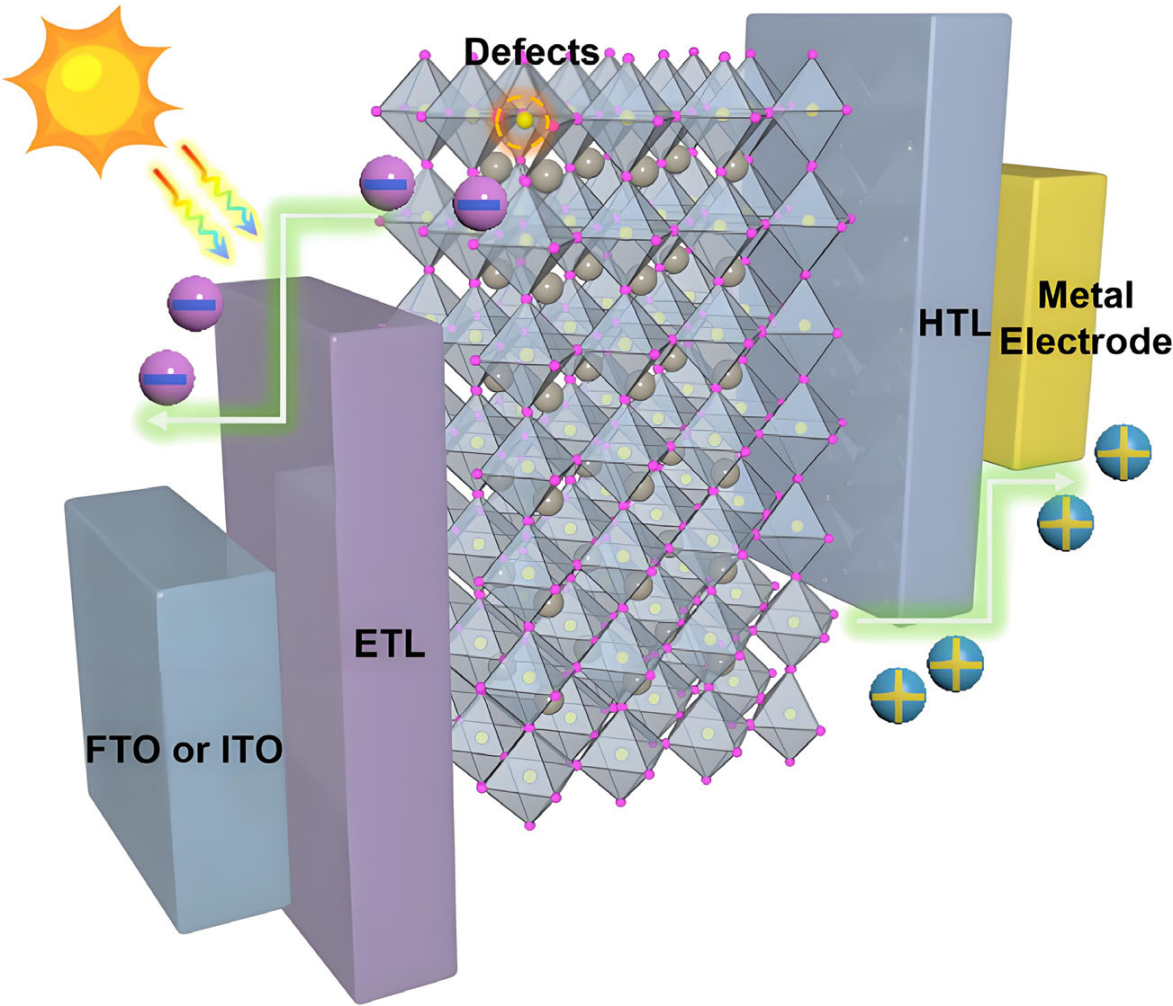
Schematic diagram of defects and ion migration in perovskite.

However, solution‐processed methods involving rapid crystallization and thermal annealing often introduce various defects in the perovskite bulk, surface, and grain boundaries (GBs), typically including undercoordinated lead (II) ions (Pb^2+^) and A‐site cations vacancies.^[^ ^7^, ^8^ ^]^ These defects not only induce nonradiative recombination of photogenerated charge carriers but also facilitate ion migration, leading to PCE degradation and impacting both photovoltaic performance and long‐term stability (**Figure** **1**).^[^ ^9^, ^10^ ^]^ Strategies such as interfacial engineering and bulk doping have been proven effective in passivating perovskite defects and suppressing nonradiative recombination loss.^[^ ^11^, ^12^, ^13^, ^14^, ^15^ ^]^

Studies have shown that introducing excess lead iodide (PbI_2_), metal cations (e.g., Cs^+^, Rb^+^, Li^+^),^[^ ^16^, ^17^, ^18^ ^]^ or halide anions (e.g., I^−^, Br^−^,^[^ ^19^ ^]^ Cl^−^,^[^ ^20^ ^]^ F^−^^[^ ^21^ ^]^) to fill vacancy defects in the perovskite lattice is a widely researched defect‐control strategy. Additionally, incorporating 2D perovskites directly into 3D perovskite systems has proven effective for defect passivation.^[^ ^22^ ^]^ Nevertheless, treatment with Lewis acid or base molecules offers another promising route. These molecules form covalent coordination bonds (acid‐base complexes) with unsaturated coordination sites (e.g., defect sites possessing lone‐pair electrons), thereby passivating defects.^[^ ^23^ ^]^ It has been reported that numerous Lewis acid/base molecules—particularly those containing N, O, or S atoms in functional groups—typically exhibit varying degrees of electron‐withdrawing character.^[^ ^24^ ^]^ Ge et al.^[^ ^25^ ^]^ reported that incorporating a nonconjugated ammonium passivator with strong electron‐withdrawing ability can modulate the heavy n‐type characteristics of low‐bandgap perovskite films, significantly enhancing the electron extraction capability of the film. Li et al.^[^ ^26^ ^]^ employed the organic dye AQ310 as a passivator, where the AQ310‐COOH groups coordinated with undercoordinated Pb^2+^, minimizing trap states and prolonging carrier recombination times.

As modifying groups, electron‐withdrawing groups (EWGs) optimize intramolecular charge distribution and electron cloud density, thereby facilitating coordinative interactions with Pb^2^^+^ ions to form more stable complexes.^[^ ^27^, ^28^, ^29^ ^]^ For example, Jiang et al.^[^ ^30^ ^]^ found that the electron‐withdrawing nature of certain molecules increases the effectiveness of passivation, thereby enhancing the overall performance of perovskite devices. The introduction of a bifunctional cyano‐containing molecule, succinonitrile (SN), into the perovskite bulk has been demonstrated to effectively coordinate Pb^2+^ defects through its two cyano (–CN) groups, which exhibit strong electron‐withdrawing properties.^[^ ^28^ ^]^ Furthermore, EWGs inherently possess a strong dipole moment. Their incorporation into various layers of PSCs enhances charge carrier extraction and transport, and they have been demonstrated as high‐efficiency modifying groups for improving both the photovoltaic performance and stability of these devices.^[^ ^31^, ^32^, ^33^, ^34^ ^]^

The use of EWGs for passivation in PSCs is a complex topic, as the chemical environment, molecular characteristics, and interactions with other species can significantly influence the outcomes. Unfortunately, the literature on electron‐withdrawing passivation in photovoltaic devices remains limited, and the working mechanisms of common electron‐withdrawing passivating materials are not yet fully clarified. This review aims to provide a comprehensive overview of the role of EWGs in PSCs, with a particular emphasis on their role in interface passivation, specifically at the electron transport layer (ETL) and hole transport layer (HTL) interfaces. We discuss the challenges faced by PSCs and provide an outlook on their future commercialization prospects, offering guidance for future research aimed at improving the optoelectronic performance of these devices through the use of EWGs.

## 2 The Principle of the Impact of Electron‐Withdrawing Molecules on the Performance of PSCs

EWGs exert their electronic effects through polarized bonds and conjugation mechanisms. Specifically, the electron‐withdrawing inductive effect refers to the phenomenon in which atoms or substituents within a molecule attract electron density toward themselves along the direction of chemical bonds. This effect induces a redistribution of the electron cloud density across the entire molecule, with the most pronounced alterations occurring near the EWGs. The perturbation propagates through electrostatic induction along the molecular chain, gradually diminishing with distance and typically vanishing beyond three carbon atoms.

**Figure 2:**
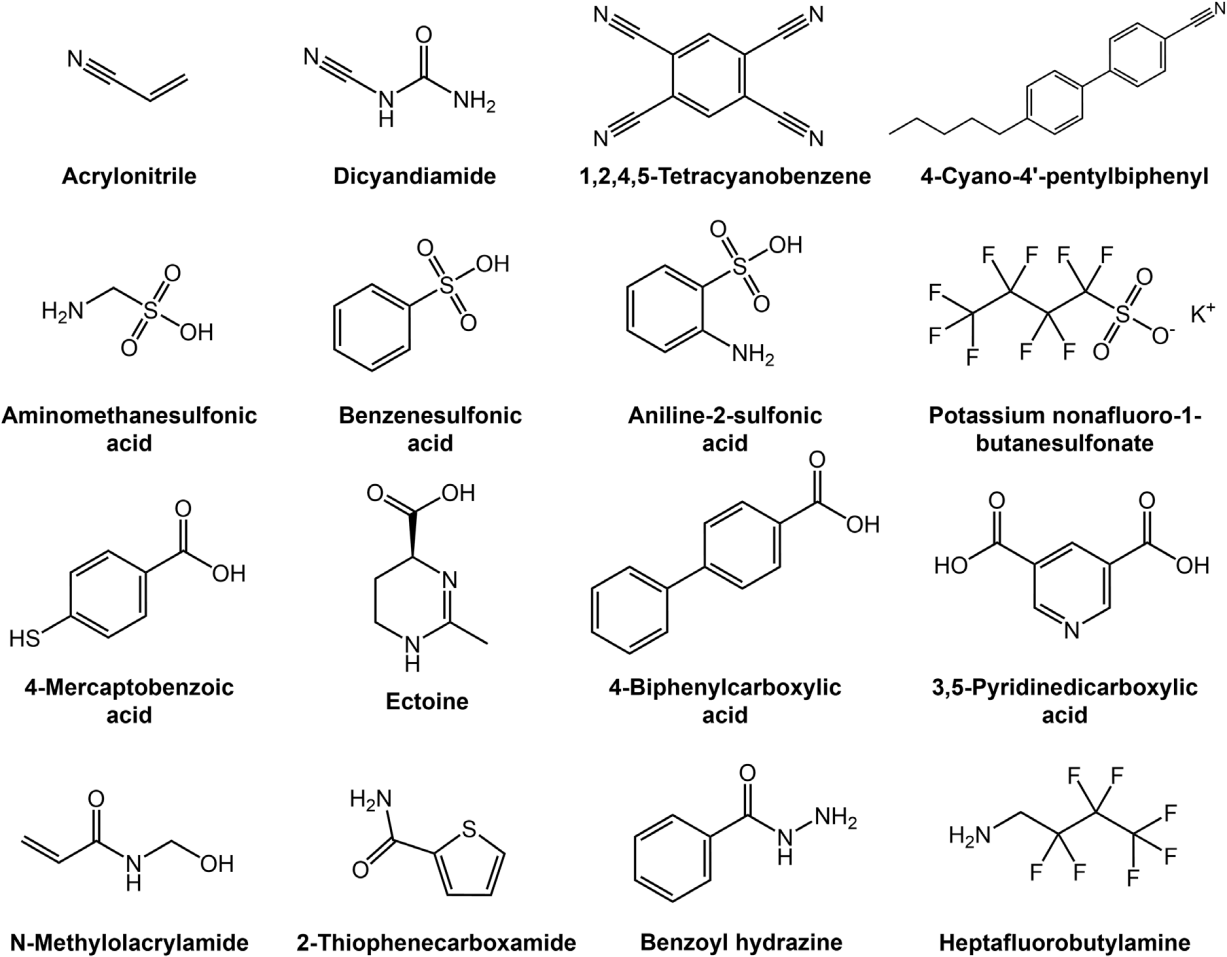
Summary of the commonly used EWMs in PSCs.

Common EWGs include –CN,^[^ ^35^ ^]^ sulfonic acid (–SO_3_H),^[^ ^32^ ^]^ carbonyl (–C=O),^[^ ^36^, ^37^, ^38^ ^]^ halogens,^[^ ^39^, ^40^, ^41^ ^]^ and others (**Figure** **2**). Notably, the strength of the electron‐withdrawing inductive effect depends on the electronegativity and atomic size of the directly bonded atom. Atoms directly connected to carbon atoms exhibit a decrease in electron‐withdrawing induction effect as the atomic number of the family increases, while increases from left to right in the same period, following: −F > −Cl > −Br > −I, −F > −OR > −NR_2_ > −CR_3_, and –OR > −SR. This systematic variation arises from differences in electronegativity and electrostatic inductive effects, which govern the ability of substituents to polarize electron density in molecular systems.

### 2.1 Defect Passivation Ability

**Figure 3:**
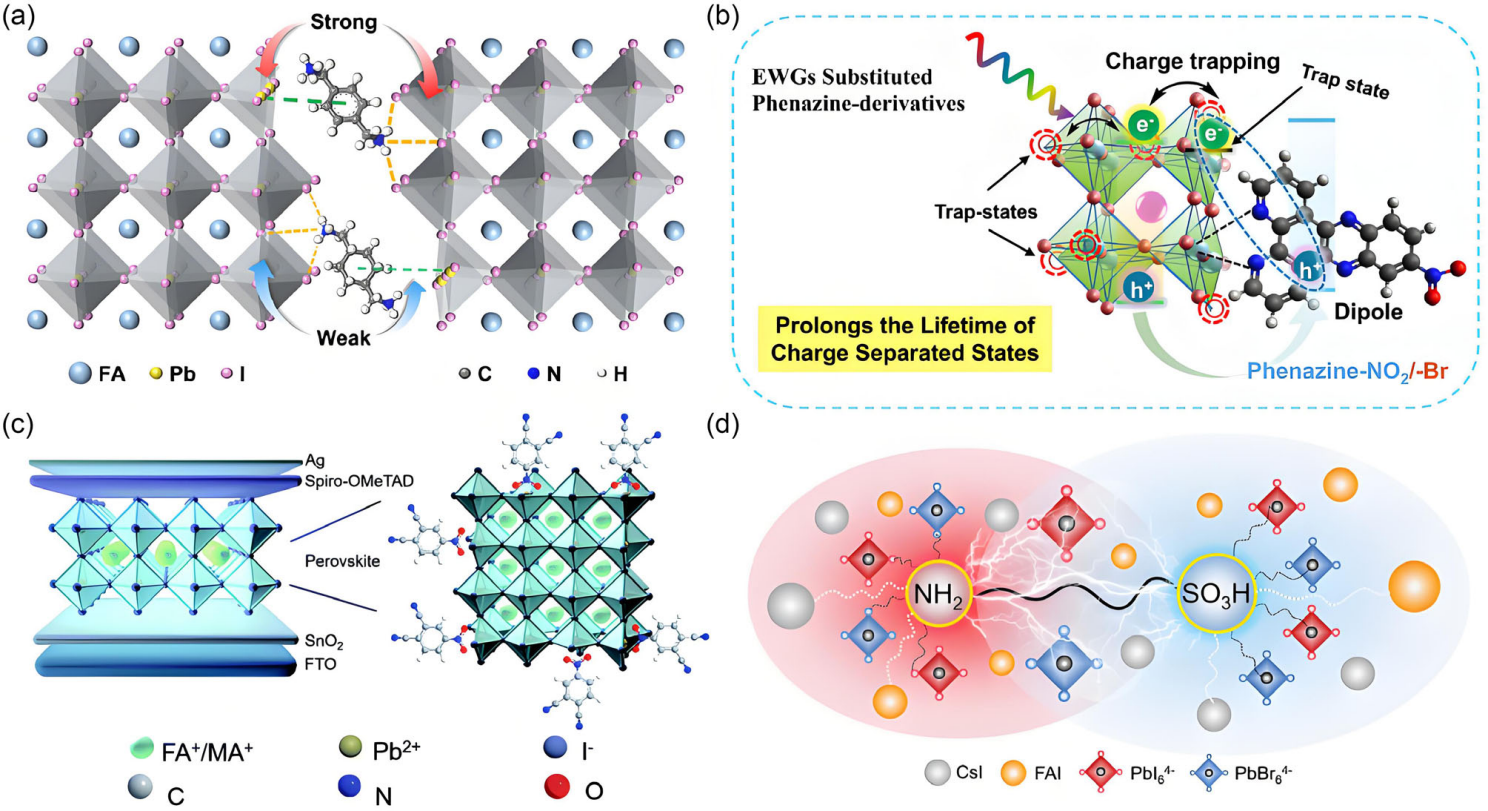
a) Schematic diagram of CyDMA^2+^ and PhDMA^2+^ passivation mechanism in the perovskite film.^[^ ^43^ ^]^ Copyright (2025), with permission from Wiley‐VCH GmbH. b) Schematic representation of excited‐state charge transfer dynamics at phenazine derivatives interface.^[^ ^45^ ^]^ Copyright (2025), with permission from Wiley‐VCH GmbH. c) Schematic illustration of the architecture of the PSCs device and the passivation mechanism of 4NPN with undercoordinated Pb^2+^ cations.^[^ ^28^ ^]^ Copyright (2021), with permission from Royal Society of Chemistry. d) Schematic diagram of zwitterionic surfactant molecule coordination with each component.^[^ ^50^ ^]^ Copyright (2023), with permission from Wiley‐VCH GmbH.

EWGs regulate defect passivation by enhancing local electronegativity, inducing interface dipole effects, and directly adjusting electric field distribution.^[^ ^42^ ^]^ EWGs are primarily employed to passivate positively charged defects in perovskite materials, such as undercoordinated Pb^2+^. Zhang et al.^[^ ^29^ ^]^ employed *p*‐phenylenediamine (PPE), terephthalonitrile (TPN), and 4‐aminobenzonitrile (ABN), containing both amino (–NH_2_) and –CN groups as passivation molecules. They revealed the role of electron density distributions on the effect of passivation, and the addition of ABN led to the best efficiency. Concurrently, EWGs polarize charge distribution; for example, phenylene‐substituted diammonium ligands enhance electron cloud density at specific sites, reducing the formation energy of lead/iodide vacancy defects and minimizing carrier scattering (**Figure** **3**a).^[^ ^43^ ^]^

### 2.2 Molecular Alignment and Mobility Enhancement

EWGs strengthen intermolecular electrostatic interactions to promote ordered π–π stacking. Such ordered alignment reduces lattice strain and enhances carrier mobility.^[^ ^44^ ^]^ For example, Aggarwal et al.^[^ ^45^ ^]^ demonstrated that methoxy‐functionalized phenazine derivatives enhance charge carrier mobility by 85.17%. This promotes dipole formation at perovskite defect sites, prolongs charge‐separated state lifetimes, and reduces nonradiative recombination losses (Figure 3b). Jiang et al.^[^ ^46^ ^]^ passivated the perovskite film with cyclohexane 1,4‐diammonium diiodide (CyDAI_2_). The *cis*‐CyDAI_2_ passivation treatment reduces the quasi‐Fermi‐level splitting–open‐circuit voltage (V_OC_) mismatch of the wide‐bandgap pero‐SCs with a bandgap of 1.88 eV and enhances its V_OC_ to 1.36 V.

### 2.3 Energy‐Level Alignment and Charge Extraction

EWGs modulate the energy band distribution and work function of perovskite thin films. By inducing pronounced band bending between the surface and bulk regions, EWGs enable spontaneous electron flow from the bulk toward the surface, thereby enhancing electron extraction and reducing carrier recombination.^[^ ^25^ ^]^ For instance, Jiang et al. demonstrated that fluorinated molecules (e.g., 2,3,5,6‐tetrafluoro‐2,5‐cyclohexadiene‐1,4‐diylidene dimalononitrile) optimize energy‐level alignment at the perovskite/HTL interface via their strong electron‐withdrawing capability, suppressing nonradiative electron‐hole recombination and extending carrier lifetimes.^[^ ^47^ ^]^ Gao et al.^[^ ^28^ ^]^ shown that strong electron‐withdrawing character lowers the lowest unoccupied molecular orbital level of 4‐nitrophthalonitrile (4NPN), optimizing energy‐level alignment between perovskites and charge transport layers (Figure 3c).

### 2.4 Enhancing Device Stability

Perovskite materials are prone to a series of thermal degradation reactions under high‐temperature conditions, such as the decomposition of the perovskite structure (e.g., PbI_2_ precipitation, halide loss).^[^ ^48^ ^]^ EWGs can help slow down these degradation processes. EWGs can strengthen the coordination between halide ions (such as I^−^ and Br^−^) and the metal center, suppress photoinduced or electric field driven halide ion migration, thereby improving the structural stability of the perovskite and reducing phase separation.^[^ ^49^ ^]^ Chen et al.^[^ ^50^ ^]^ found that introducing EWG can enhance the binding energy of halide ions and slow down their volatilization and precipitation at high temperatures (Figure 3d). Moreover, EWGs can reduce the electron density of metal cations (such as Pb^2+^ or Sn^2+^), making them more stable.^[^ ^51^, ^52^ ^]^ This effectively prevents metal cation migration or oxidation at high temperatures, thus reducing thermal degradation of the perovskite material.

## 3 The Application of Electron‐Withdrawing Passivation Materials in PSCs

**Figure 4:**
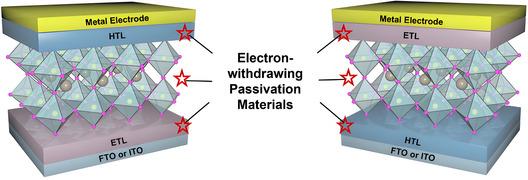
Schematic diagram of modifying perovskite bulk and interface of PSCs with EWMs.

As mentioned above, electron‐withdrawing passivation materials can be widely applied in PSCs. It is certain that different passivation materials work in different ways. Wu et al.^[^ ^53^ ^]^ report an amphiphilic molecular hole transporter, (2‐(4‐(bis(4‐methoxyphenyl)amino)phenyl)‐1‐cyanovinyl) phosphonic acid, that features a multifunctional cyanovinyl phosphonic acid group and forms a superwetting underlayer for perovskite deposition, which enables high‐quality perovskite films with minimized defects at the buried interface. Jiang et al.^[^ ^30^ ^]^ found that the π‐electrons of aromatic units in conjugated systems do not directly demonstrate strong passivation towards defects. Instead, they enhance the electron cloud density of EWGs, thereby strengthening their interaction with defect sites. Zhang et al.^[^ ^54^ ^]^ employed electron‐withdrawing materials (EWMs) to induce stronger interface coupling at the TiO_2_/PbI_2_ interface, which immobilized PbI_2_ on the surface and consequently improved device performance. This chapter focuses on the application of electron‐withdrawing passivation materials in PSCs, referencing different interface layers and summarizing the working mechanisms of each type of passivation material, as well as their impact on device performance (**Figure** **4**).

### 3.1 Electron‐Withdrawing Passivation Materials for Perovskite

EWGs play key roles in the optimization of perovskite absorber layers, with functions including defect passivation, carrier dynamics control, interface energy‐level control, lattice stress suppression, and stability enhancement. The representative EWGs based on electron‐withdrawing passivation materials for perovskite are summarized in **Table** **1**.

**Table 1:** Device performance of PSCs prepared by perovskite layer modified with different EWMs.

EWM	Device structure	V_OC_ [V]	FF [%]	J_SC_ [mA cm^−2^]	PCE [%]		Ref.
BCDT	FTO/TiO_2_/ (FAPbI_3_)_0.85_(MAPbBr_3_)_0.15_/Spiro‐OMeTAD/MoO_3_/Ag	1.150	79.0	24.44		22.20	57
DCMH	ITO/SnO_2_/FAPbI_3_/Spiro‐OMeTAD/Au	1.160	83.3	25.09		24.26	58
4Cl‐BZS	FTO/MeO‐2PACz/Cs_0.05_MA_0.1_FA_0.85_PbI_3_/C_60_/SnOx/Ag	1.180	86.2	26.40		26.90	60
ZR3	ITO/NiO_*X*_/MeO‐2PACz/ FA_0.95_Cs_0.05_PbI_3_/ZR3/C_60_/BCP/Ag	1.184	84.8	25.86		25.96	61
4FPEAPSA	ITO/MeO‐2PACz/ Rb_0.05_Cs_0.05_MA_0.05_FA_0.85_Pb[I_0.95_Br_0.05_]_3_/C_60_/BCP/Ag	1.195	84.3	24.85		25.03	64
FIPh‐A	FTO/TiO_2_/[FA_0.62_MA_0.34_Cs_0.04_]Pb[I_0.98_Br_0.02_]_3_/Spiro‐OMeTAD/Au	1.176	79.4	26.33		24.60	66
M4	ITO/MeO–2PACz/ Cs_0.05_[FA_0.95_MA_0.05_]_0.95_Pb[I_0.95_Br_0.05_]_3_/M4/PCBM/BCP/Ag	1.180	85.2	24.84		25.10	69
FPA	ITO/NiO_*X*_/MeO‐4PACz/[FA_0.95_MA_0.05_]_0.95_Pb[I_0.95_Br_0.05_]_3_ /PCBM/BCP/Ag	1.140	83.3	26.77		25.37	70
AIDCN	ITO/ NiO_*X*_/Me‐4PACz/Cs_0.25_FA_0.75_Pb[Br_0.5_I_0.5_]_3_ /PCBM/BCP/Ag	1.366	84.2	16.10		18.52	72
AMT	ITO/PEDOT:PSS/2D DJ perovskite/PCBM/BCP/Ag	1.101	77.7	22.97		19.69	75
DFAz	ITO/PTAA/2D RP perovskite/PCBM/BCP/Ag	1.090	78.2	22.61		19.28	77
CMP	ITO/PEDOT:PSS/FA_0.7_MA_0.27_Cs_0.03_Pb_0.5_Sn_0.5_I_3_/C_60_/BCP/Ag	0.852	79.8	31.82		21.65	52
AESA	ITO/NiO_*X*_/Cs_0.2_FA_0.8_Pb(I_0.6_Br_0.4_)_3_/PCBM/BCP/Ag	1.250	83.7	18.79		19.66	51
PZDI	ITO/NiO_*X*_/MeO‐4PACz/Cs_0.05_MA_0.1_FA_0.85_[Pb_0.5_Sn_0.5_]I_3_ /C_60_/BCP/Ag	1.188	78.7	24.78		23.17	45
PhDMADI	ITO/NiO_*X*_/2PACz/FA_0.85_MA_0.1_Cs_0.05_PbI_3_/C_60_/BCP/Cu	1.183	85.1	25.85		26.04	44
CCPI	FTO/IDCz‐3/FA_0.85_MA_0.1_Cs_0.05_PbI_3_/C60/BCP/Ag	1.166	85.4	25.82		25.74	25
ThFABr	ITO/SnO_2_/ MA_0.16_FA_0.84_PbI_3_/Spiro‐OMeTAD/MoO_3_/Ag	1.210	81.6	25.07		24.69	24

#### 3.1.1 Cyano Groups

**Figure 5:**
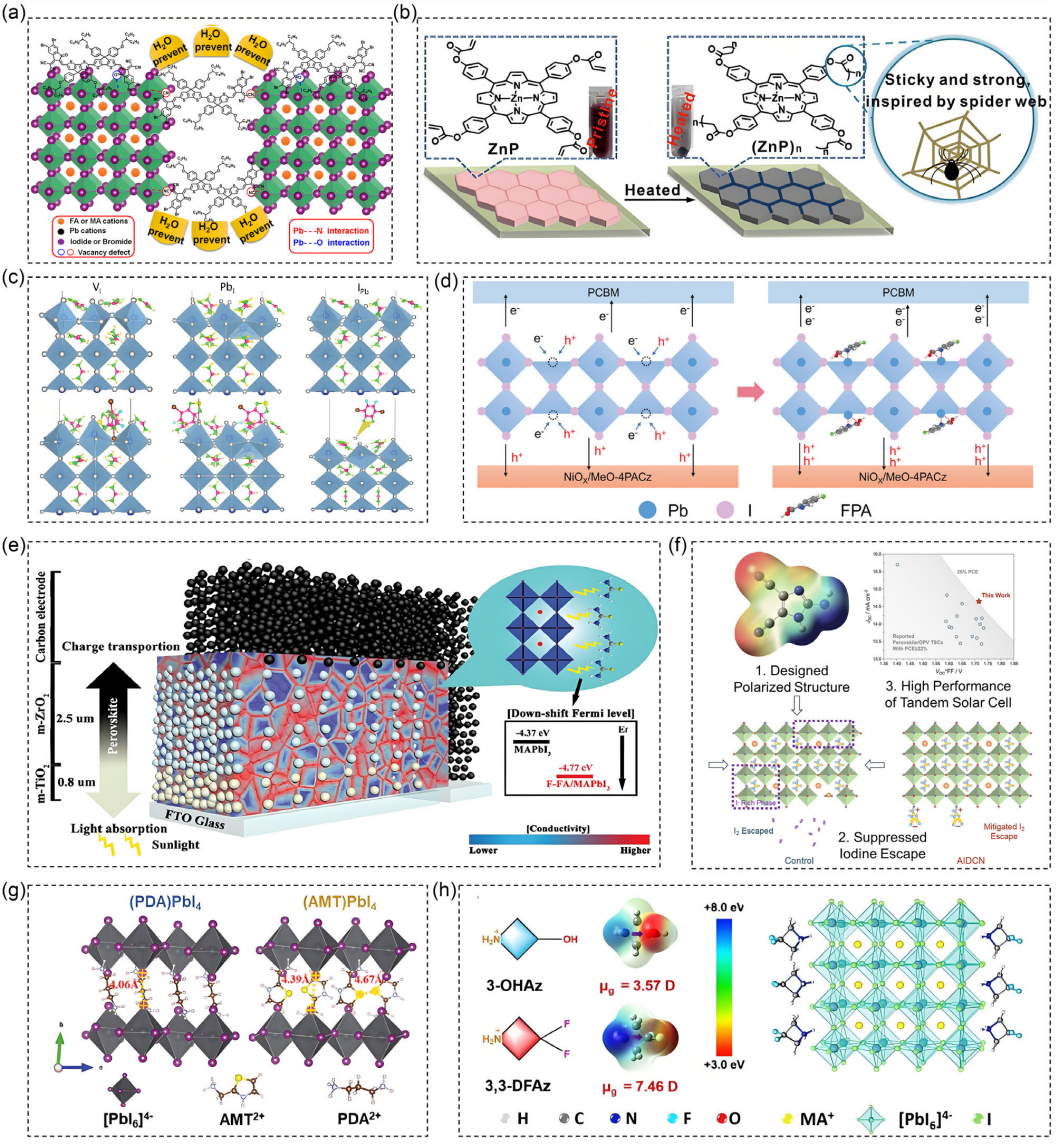
a) Schematic diagram of GB passivation by compound BCDT, which can prevent H_2_O from entering to degrade the perovskite.^[^ ^55^ ^]^ b) Perovskite film modified by ZnP.^[^ ^63^ ^]^ Copyright (2021), with permission from Elsevier. c) The effect of M4 on VI, PbI, and IPb defects in perovskite films.^[^ ^67^ ^]^ Copyright (2024), with permission from Wiley‐VCH GmbH. d) Comparative regulatory mechanisms of the control and FPA‐treated films.^[^ ^68^ ^]^ Copyright (2024), with permission from Wiley‐VCH GmbH. e) The schematic diagram of PSCs with F‐FA/MAPbI_3_ filled inside.^[^ ^69^ ^]^ Copyright (2021), with permission from Wiley‐VCH GmbH. f) The powerful electron‐withdrawing effect of AIDCN enhances the energy barrier formed by iodine vapor.^[^ ^70^ ^]^ Copyright (2024), with permission from Elsevier Inc. g) The crystal structures of (PDA)PbI_4_ (left) and (AMT)PbI_4_ (right).^[^ ^73^ ^]^ Copyright (2024), with permission from Wiley‐VCH GmbH. h) Schematic crystal structure of 3,3‐DFAz‐Pb (nominal *n* = 4).^[^ ^75^ ^]^ Copyright (2024), with permission from Wiley‐VCH GmbH.

The nitrogen atom in cyanide molecules has arc pair electrons, which can form coordination bonds with uncoordinated Pb^2+^ and reduce the density of defect states. For instance, Wu et al.^[^ ^55^ ^]^ proposed using bicyclo[2,2,1]hept‐2‐ene‐5,6‐diamine (BCDT), a compound containing –CN group, as a passivating agent. By adding BCDT to the antisolvent during film formation, the grain boundary defects were passivated, and grain growth was promoted (**Figure** **5**a).

Time resolved photoluminescence (PL) measurements confirmed that this strategy increased carrier recombination lifetimes and improved long‐term stability.^[^ ^56^ ^]^ Wolf et al.^[^ ^57^ ^]^ utilized organic cyano‐π‐conjugated molecules, specifically indeno[3,2‐b]thiophene (IDTT) containing a –CN group, to self‐anchor at the GBs of perovskite films. This approach effectively passivated uncoordinated Pb^2+^ ions, enhancing charge separation and transport at the GBs.

The partially positively charged carbon atoms of the cyanide group can form weak hydrogen bonds with adjacent organic cations, which helps stabilize the organic cations at the surface and GBs. Shao et al.^[^ ^34^ ^]^ introduced succinonitrile (–SN), a bidirectional cyano molecule with two electron‐withdrawing –CN groups at both ends of the carbon chain, to the perovskite precursor solution. SN was found to effectively coordinate with uncoordinated Pb^2+^ ions, forming hydrogen bonds with –NH_2_ groups and suppressing trap‐assisted nonradiative carrier recombination.

#### 3.1.2 Sulfonic Acid Groups

Sulfonic acid groups have strong acidity and are prone to releasing protons to passivate iodine vacancies. At the same time, the sulfonic acid ions (–SO_3_^−^) generated after dissociation usually form bidentate or even multidentate coordination bonds with one or more uncoordinated Pb^2+^ ions to passivate defects.^[^ ^58^ ^]^ Hua et al.^[^ ^59^ ^]^ employed 4,4'‐(1,4‐phenylenebis(oxy))bis(butane‐1‐sulfonic acid sodium salt) (ZR3), a multifunctional modifier containing sulfonate groups (SO_3_^−^) and Na^+^ ions, to treat perovskite films. This strategy enhanced exciton dissociation in the perovskite layer and improved energy‐level alignment with the HTL, thereby optimizing charge extraction efficiency. Jiang et al.^[^ ^60^ ^]^ improved the uniformity of the film, promoted larger grain domains, and facilitated more efficient charge transfer through GBs by passivating grain boundary defects with sodium polystyrene sulfonate.

The strong acid‐base reaction between sulfonic acid groups and organic cations generates stable ammonium sulfonate salts, which strongly inhibit cation migration and volatilization, and improve film stability. Zou et al.^[^ ^61^ ^]^ introduced the anionic surfactant sodium dodecylbenzenesulfonate (SDBS) to simultaneously passivate defect states and stabilize the cubic phase of perovskite films. SDBS located at GBs and active layer surfaces can effectively passivate lead ions with insufficient coordination, protecting perovskite components from water induced degradation. Tan et al.^[^ ^62^ ^]^ designed a amphoteric organic salt, 2‐(4‐fluorophenyl) ethylammonium‐4‐methylbenzenesulfonate (4FPEAPSA), to optimize the film morphology and energy‐level arrangement of perovskite buried at the interface. 4FPEAPSA treatment promotes the growth of pore free, coarse‐grained hydrophobic thin films by inducing crystal orientation. In addition, the dual functional 4FPEAPSA can passivate defects of iodine and methylamine vacancies, reducing defect density.

#### 3.1.3 Carbonyl Groups

The defects in carbonyl modified perovskite mainly rely on the strong electronegativity of its oxygen atoms. The common uncoordinated Pb^2+^ lacks sufficient I^−^ coordination, and its empty sp/d orbitals easily accept lone‐pair electrons from carbonyl oxygen atoms to form coordination bonds. Cao et al.^[^ ^63^ ^]^ employed in situ polymerization of zinc porphyrin (ZnP) with a carbonyl group, which successfully fixed lead within the perovskite lattice. This encapsulation of the perovskite film suppressed lead leakage, reduced trap densities, and minimized nonradiative recombination, as confirmed by PL and electrochemical impedance spectroscopy measurements (Figure 5b). Wang et al.^[^ ^64^ ^]^ designed a multifunctional molecule, N_1_,N_4_‐bis(2,3,5,6‐tetrafluoro‐4‐iodophenyl)terephthalamide (FIPh‐A), which contains electron‐withdrawing carbonyl and tetrafluorophenyl iodide groups. This molecule stabilizes Pb^2+^ ions and the [PbI_6_]^4−^ octahedral structure, thus preventing ion migration within perovskite films. Grätzel et al.^[^ ^65^ ^]^ introduced an anion engineering concept, using the pseudohalide anion formate (HCOO−) to suppress anion‐vacancy defects presented at GBs and the surface of the perovskite films to augment the crystallinity of the films.

#### 3.1.4 Halogen Groups

Introducing halogen groups (Br^−^, Cl^−^, etc.) can fill iodine vacancy defects and form coordination with uncoordinated Pb^2+^, reducing defect density. In addition, hydrophobic halogen groups (F) can also improve the stability of the film and prevent water and oxygen erosion.^[^ ^66^ ^]^ Li et al.^[^ ^67^ ^]^ introduced an organic molecule, 4,7‐bromo‐5,6‐fluoro‐2,1,3‐phenylpropylthiazole (M4), which contains electron‐withdrawing bromine (Br) and fluorine (F) sites. The Br sites interact with surface lead (Pb) atoms, repairing iodine vacancy defects, while the F sites correct octahedral lattice distortions and eliminate Pb defects (Figure 5c). Wang et al.^[^ ^68^ ^]^ proposed a dual‐interface modification strategy using 5‐fluoropyridine acid (FPA) to regulate crystal growth and passivate defects. The electron‐withdrawing carbonyl groups in FPA interact with uncoordinated Pb^2+^/Pb clusters, stabilizing the Pb—I framework. In situ PL measurements confirmed that the treated films exhibited lower PL intensity and shorter carrier lifetimes (decreasing from 14.97 to 6.38 ns), indicating the formation of high‐quality perovskite films (Figure 5d).

Han et al.^[^ ^69^ ^]^ synthesized a new N,1‐fluoroformamidinium iodide (F‐FAI) cation, where a fluorine electron‐withdrawing group replaced an amino group in guanidinium (GA^+^), and used it as an additive in PSCs. The electron‐withdrawing effect of F enhanced the molecular polarity of F‐FAI, improving the interaction between F‐FAI and MAPbI_3_ (Figure 5e). Ning et al.^[^ ^51^ ^]^ developed an electron‐withdrawing chloromethylphosphonic acid ligand (CMP). The complex formed between SnI_2_ and CMP exhibited a deeper highest occupied molecular orbital (HOMO) level, improved redox potential, and a significantly increased ionization potential of the perovskite structure. This work alleviated the oxidation issue and enabled the fabrication of a fully perovskite tandem solar cell with a certified efficiency of 26.96%.

#### 3.1.5 Others

The synergistic effect between EWGs enhances their interaction with defects. Hou et al.^[^ ^70^ ^]^ designed 2‐amino‐4,5‐imidazole‐2‐carboxylic nitrile (AIDCN), a bifunctional molecule with a synergistic effect of carboxyl and cyanide groups. Grazing incidence wide‐angle X‐ray scattering and X‐ray fluorescence measurements indicate that doping AIDCN into perovskite films can suppress photoinduced iodine loss. The strong electron‐withdrawing effect of AIDCN, combined with its highly polarized charge distribution, enhances the energy barrier for iodine vapor formation and reduces halide phase segregation (Figure 5f). EWMs are also widely used in 2D layered perovskites.^[^ ^71^, ^72^ ^]^ For example, Chen et al.^[^ ^73^ ^]^ introduced 2‐thiazolylmethylammonium (AMT), which contains a thiazole core, into perovskite precursor solutions to form 2D Dion–Jacobson (DJ) perovskites. AMT is reported to possess delocalized π‐electrons and a strong electron‐withdrawing ability. The perovskite films based on AMT exhibit a type II quantum well structure, which is beneficial for exciton dissociation. Density functional theory calculations show strong orbital coupling between AMT and the inorganic perovskite layers (Figure 5g). Similarly, Zhang et al.^[^ ^74^ ^]^ employed ethyl acetate (EA), a green antisolvent containing an electron‐withdrawing carbonyl group, to achieve uniform phase dispersion in quasi‐2D DJ‐phase perovskite films (DMePDA) FA_3_Pb_4_I_13_ without the use of methylammonium (MA). The organic spacer groups in these films optimize both the structural and optical properties of the layered 2D perovskites. Liu et al.^[^ ^75^ ^]^ developed a spacer material, 3,3‐difluoro‐1,2,3,4‐tetrahydropyrrolo[3,4‐b]quinoxaline (3,3‐DFAz), which contains various EWGs. This spacer reduces exciton binding energy, leading to high‐efficiency 2D perovskites with a PCE of 19.28% (Figure 5h).

Future research should prioritize the rational molecular design of multifunctional EWMs to simultaneously suppress ionic migration, eliminate deep‐level traps, and enhance phase stability through strong host‐guest interactions. Furthermore, scalable deposition methods require development for uniformly integrating EWMs into large‐area, thick perovskite films while maintaining homogeneity, thereby bridging lab‐scale efficiency gains with commercial viability.

### 3.2 Electron‐Withdrawing Passivation Materials for HTL

Doping electron‐withdrawing passivation materials into the HTL can also effectively passivate defects and regulate band bending (**Table** **2**). Liu et al.^[^ ^76^ ^]^ synthesized carbazole‐based molecules (JY7) and molecules with different EWGs, such as fluorine and cyano (JY8 and JY9), and incorporated them into the hole transport material. Molecular dynamics simulations and experimental results indicated that the introduction of EWGs increased the HOMO energy levels, enhancing interface adsorption capabilities. Wang et al.^[^ ^77^ ^]^ reported that trimethoxysilane with EWGs, generates molecular dipoles directed toward the HTL, altering the HTL's work function and optimizing energy‐level alignment. This provides a convenient strategy for designing interfacial modification materials. Alex et al.^[^ ^78^ ^]^ synthesized a novel DA‐D type hole transport material with electron‐withdrawing cyanide groups (BTF5 and BTF6). They found that compared to BTF5 with para methoxy substitution, BTF6 with *meta* methoxy substitution exhibited more matching energy levels with perovskite and greatly enhanced hole mobility.

**Table 2:** Device performance of PSCs prepared by HTL layers modified with different EWMs.

EWM	Device structure	V_OC_ [V]	FF [%]	J_SC_ [mA cm^−2^]	PCE [%]	Ref.
JY8	FTO/TiO_2_/Cs_0.05_FA_0.85_MA_0.10_Pb[I_0.88_Br_0.04_Cl_0.08_]_3_/JY8/Ag	1.131	81.0	24.58	22.53	78
F_3_‐TMOS	FTO/PEDOT:PSS/F_3_‐TMOS/FASnI_3_/ICBA/BCP/Ag	0.910	71.5	22.52	14.67	79
BTF6	ITO/BTF6/[FA_0.92_MA_0.08_]_0.9_Cs_0.1_Pb[I_0.92_Br_0.08_]_3_/C60/BCP/Ag	1.130	81.0	22.23	20.30	80
FN‐S	FTO/SnO_2_/FAPbI_3_/FN‐S/Au	1.160	81.0	24.83	23.25	81
TA	ITO/SnO_2_/ Cs_0.05_FA_0.95_PbI_3_/spiro‐OMeTAD(TA)/Au	1.146	80.2	24.49	22.52	82
COF	ITO/SnO_2_/ Cs_0.03_FA_0.97_PbI_3_/spiro‐OMeTAD(COF)/Au	1.180	80.6	25.39	24.25	83
MSBH	ITO/SnO_2_/ Cs_0.07_FA_0.93_PbI_3_/Spiro‐OMeTAD(MSBH)/MoO_3_/Ag	1.190	80.9	26.31	25.32	84
MPA‐CPA	ITO/NiO_*X*_/MPA‐CPA/Cs_0.05_[FA_0.95_MA_0.05_]_0.95_Pb[I_0.95_Br_0.05_]_3_/C_60_/BCP/Ag	1.201	84.5	24.80	25.16	54
TBT	ITO/NiO_*X*_/TBT/Cs_0.04_[FA_0.96_MA_0.04_]_0.96_Pb[I_0.96_Br_0.04_]_3_ /PEAI/PCBM/BCP/Ag	1.190	83.7	24.90	24.80	86
CBSA	ITO/NiO_*X*_/CBSA/Cs_0.05_(FA_0.92_MA_0.08_)_0.95_[I_0.92_Br_0.08_]_3_/PCBM/BCP/Ag	1.110	81.4	24.01	21.80	87
TFSA	TO/NiO_*x*_/MeO‐2PACz(TFSA)/ Cs_0.05_FA_0.95_PbI_3_/C_60_/BCP/Ag	1.169	85.1	26.07	25.92	88
PTTI‐TPA	ITO/PTTI‐TPA/[FA_0.17_MA_0.94_PbI_3.11_]_0.95_[PbCl_2_]_0.05_/C_60_/BCP/Ag	1.100	83.3	22.99	21.00	90
Taurine	ITO/Taurine/[FASnI_3_]_0.6_[MAPbI_3_]_0.4_/C_60_/BCP/Cu	0.911	77.1	32.02	22.50	91

**Figure 6:**
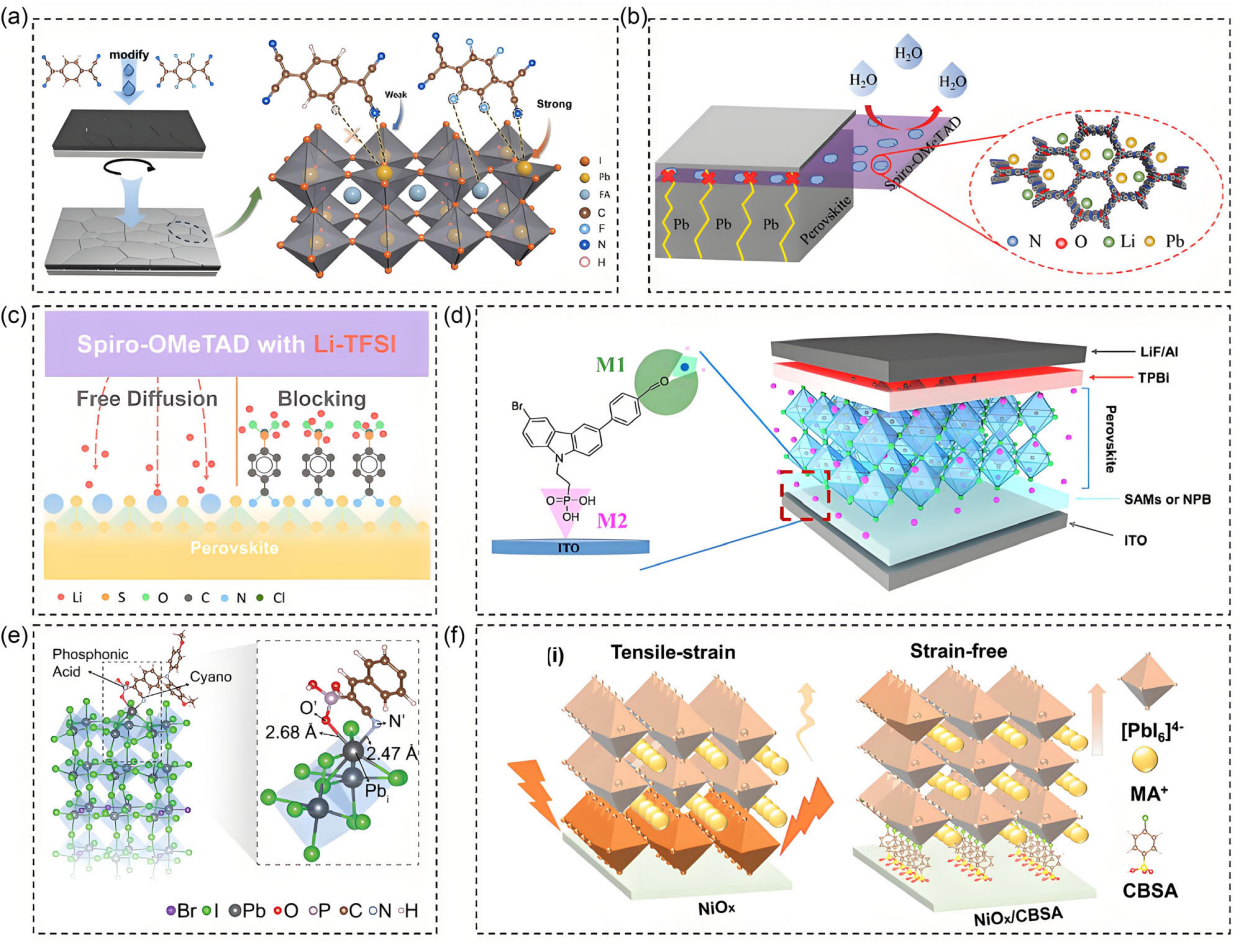
a) Schematic diagram of the interaction between TCNQ and F4TCNQ with defects.^[^ ^47^ ^]^ b) Schematic description of the role of COF in HTL and its effect on suppressing Pb leakage.^[^ ^81^ ^]^ Copyright (2024), with permission from Wiley‐VCH GmbH. c) Schematic diagram of MSBH blocking Li‐ions’ migration.^[^ ^82^ ^]^ Copyright (2024), with permission from American Chemical Society. d) Schematic diagram of CZPC in different HTLs.^[^ ^83^ ^]^ Copyright (2023), with permission from Elsevier. e) Multifunctional cyanoethylenephosphonic acid groups optimize structure of the passivated surface.^[^ ^53^ ^]^ Copyright (2023), with permission from the American association for the advancement of science. f) Schematic diagrams of lattice strain distribution in perovskite grains deposited on NiO_*X*_ and NiO_*X*_/CBSA substrates.^[^ ^85^ ^]^ Copyright (2022), with permission from Wiley‐VCH GmbH.

Zhu et al.^[^ ^79^ ^]^ synthesized new organic molecules with EWGs, such as thiocarbonyl (C=S) and carbonyl (C=O) functional groups, named FN—S and FN—O, and applied them to HTMs. The sulfur atoms in FN—S have a larger negative charge distribution than the oxygen atoms in FN—O, exhibiting more suitable energy levels and thus possessing excellent hole extraction and transport capabilities. Jiang et al.^[^ ^47^ ^]^ compared the effects of 2,2'‐(2,5‐cyclohexadiene‐1,4‐diyl)bis(malonitrile) (TCNQ) and (2,3,5,6‐tetrafluoro‐2,5‐cyclohexadiene) on the perovskite/HTL interface. PL quantum yield measurements confirmed that F4TCNQ‐modified perovskite films exhibited greater quasi‐Fermi level splitting (ΔEF) compared to other samples. The ordered arrangement of electron‐withdrawing functional groups generates an electric dipole effect, interacting with Pb ions on the perovskite film's surface and creating a permanent dipole moment, which reduces trap state density (**Figure** **6**a).

The 2,2',7,7'‐Tetrakis[N,N‐di(4‐methoxyphenyl)amino]‐9,9'‐spirobifluorene (Spiro‐OMeTAD), as a widely used hole transport material, is extensively applied in PSCs. However, its intrinsic conductivity is relatively low, necessitating the addition of dopants such as lithium bis(trifluoromethanesulfonyl)imide (Li‐TFSI) during the procedure. Unfortunately, the aggregation of Li salts negatively impacts the conductivity of perovskite devices. To address this, Chen et al.^[^ ^80^ ^]^ incorporated a natural small‐molecule additive containing an electron‐withdrawing carbonyl group‐thioctic acid (TA)‐into Spiro‐OMeTAD. By measuring the storage modulus (G′) and loss modulus (G″), they demonstrated that TA interacts with Li salts to form a gel‐like HTL, thereby suppressing Li‐TFSI aggregation and the resulting voids in the film. Due to the strong electronegativity of the sulfur (S) atoms in TA, which exert a strong attraction on the electron cloud of the carbon (C) atoms, the material facilitates the oxidation of Spiro‐OMeTAD, enhancing its conductivity.

While Li‐TFSI can enhance HTL conductivity, its migration of lithium ions can negatively impact device stability, and doping with electron‐withdrawing passivation materials effectively addresses this issue. Park et al.^[^ ^81^ ^]^ used chemically modified 2D conjugated covalent organic frameworks (Tp‐Azo‐COF) doped into the Spiro‐OMeTAD HTL. Tp‐Azo‐COF contains abundant carbonyl (C=O) groups and azo (N=N) nodes, with excellent chelation and adsorption abilities, effectively reducing Pb leakage and lithium ion migration. The treated devices achieved an impressive efficiency of 24.25%, setting a new benchmark for COF‐modified devices (Figure 6b). Wang et al.^[^ ^82^ ^]^ used 4‐methylsulfonylbenzamidine hydrochloride (MSBH) as an additive in the HTL to suppress ionic migration, thereby controlling Li ion movement into the perovskite layer and improving device stability (Figure 6c).

In trans batteries, self‐assembled monolayers (SAMs) are often used as HTLs. SAMs modified with EWGs can not only passivate Pb^2+^ defects, but also improve the deposition of perovskite in the TCO layer. Wang et al.^[^ ^83^ ^]^ developed (2‐(3‐bromo‐6‐(4‐formylphenyl)‐9 H‐carbazole‐9‐yl)ethyl)phosphonic acid (CZPC) as a SAM, where the carbonyl (C=O) group serves as an electron‐withdrawing group. The interaction between the noncoordinating Pb^2+^ and the electron‐withdrawing group enhances crystallinity and suppresses nonradiative losses at the buried perovskite–hole transport interface (Figure 6d). Wu et al.^[^ ^53^ ^]^ reported an amphiphilic hole transport material, 2‐(4‐(bis(4‐methoxyphenyl)amino) phenyl)‐1‐cyanoethylenephosphonic acid, which features multifunctional cyanoethylenephosphonic acid groups, forming a superwetting underlying layer for perovskite deposition and achieving a certified PCE of 25.4% (Figure 6e).

Electron‐withdrawing passivation materials can also regulate the contact and carrier transport between SAM and NiO_*X*_. For example, Zhou et al.^[^ ^84^ ^]^ used a series of methoxy‐substituted triphenylamine‐functionalized benzothiadiazole (TBT)‐based SAM molecules to modify NiO_*X*_, enhancing charge collection and suppressing interface reactions. Additionally, Chen et al.^[^ ^85^ ^]^ employed *p*‐chlorobenzenesulfonic acid (CBSA) SAMs, where the electron‐withdrawing chlorine terminal of the SAMs provides growth sites for perovskite, enabling dual passivation of both NiO_*X*_ and perovskite crystals (Figure 6f). Peng et al.^[^ ^86^ ^]^ used electron‐withdrawing fluorine‐modified tetrafluorosuccinic acid (TFSA) to minimize energy losses at the HTL/ perovskite interface, achieving a stable inverted PSCs with an efficiency of 25.92%.

Nazeeruddin et al.^[^ ^87^ ^]^ reported several D‐π‐A molecules as hole materials, which contain rigid quinoline acridine (FA‐CN) and flexible triphenylamine (TPA‐CN) as central units and donor moieties, alkyl substituted tertiary thiophenes as conjugated bridges, and malononitrile as electron acceptor moieties. The malenonitile moiety, one of the strongest electron acceptors, can stabilize the HOMO energy level. FA‐CN and TPA‐CN, as undoped hole materials, exhibit excellent PCE of 18.9% and 17.5%, respectively, under sunlight irradiation. Guo et al.^[^ ^88^ ^]^ developed two acylated triarylamine‐based donor‐type copolymers, PBTI‐TPA and PTTI‐TPA, as undoped HTLs in inverted PSCs. The combination of a classic redox‐active triphenylamine donor unit and an electron‐withdrawing oligothiophene imide co‐unit with rigid and planar backbone furnishes the two polymers with quasiplanar backbone, suitable frontier molecular orbital energy levels, favorable thermal stability, appropriate film morphology, and passivation effect. Wang et al.^[^ ^89^ ^]^ replaced the traditional HTL (PEDOT:PSS), with 2‐aminoethanesulfonic acid as an interface bridge. This modification improved the optoelectronic performance of the perovskite absorber at the buried contact, and the ITO substrate modified with 2‐aminoethanesulfonic acid showed lower optical losses, significantly enhancing the V_OC_ of the device.

Future research should focus on designing energy‐level‐tunable EWMs to synergistically optimize interfacial band alignment while suppressing recombination at the perovskite/HTL junction. Critical challenges include balancing hole extraction efficiency with interfacial defect passivation, as excessive electron affinity may impede charge selectivity. Future studies need to elucidate the dynamic interactions between EWMs, perovskite surfaces, and environmental factors to guide molecular engineering strategies.

### 3.3 Electron‐Withdrawing Passivation Materials for ETL

In traditional n‐i‐p PSCs, the bottom surface of the perovskite film is usually in direct contact with the ETL. Common ETL materials include transparent n‐type semiconductors such as titanium dioxide (TiO_2_) and tin oxide (SnO_2_).^[^ ^90^ ^]^ The ETL plays a crucial role in rapidly extracting photogenerated electrons in PSCs (**Table** **3**). Due to the energy level differences between materials and thermodynamic effects, photogenerated electrons from the perovskite conduction band are injected into the conduction band of the ETL. Introducing suitable materials to adjust the energy‐level alignment can enhance this electron transfer capability.

**Table 3:** Device performance of PSCs prepared by ETL layers modified with different EWMs.

EWM	Device structure	V_OC_ [V]	FF [%]	J_SC_ [mA cm^−2^]	PCE [%]	Ref.
KFBS	ITO/SnO_2_/KFBS/FA_0.9_MA_0.1_PbI_3_/Spiro‐OMeTAD/Ag	1.144	81.7	24.83	23.21	95
NOBF_4_	ITO/SnO_2_/NOBF_4_/FA_0.9_MA_0.1_PbI_3_/Spiro‐OMeTAD/Au	1.148	82.9	25.24	24.04	99
SK	FTO/TiO_2_/SK/FAPbI_3_/Spiro‐OMeTAD/Au	1.165	84.9	25.51	25.22	100
OA	FTO/SnO_2_/OA/FAPbI_3_/Spiro‐OMeTAD/Au	1.160	83.2	25.60	24.60	101
BTPC_60_	ITO/MeO‐2PACz/Cs_0.05_[FA_0.98_MA_0.02_]_0.95_Pb[I_0.98_Br_0.02_]_3_/BTPC_60_/BCP/Ag	1.180	84.0	25.50	25.30	105
CBCM	ITO/NiO*x*/Me‐4PACz/Cs_0.05_[FA_0.98_MA_0.02_]_0.95_PbI_3_/CBCM/BCP/Ag	1.183	84.4	25.93	25.89	106
FP‐C8	ITO/PTAA/PFN‐Br/ Cs_0.05_FA_0.90_MA_0.05_PbI_2.85_Br_0.15_/FP‐C8/BCP/Ag	1.120	83.4	24.65	23.08	107
C3N	FTO/SnO_2_/C3N/[FAPbI_3_]_0.992_[MAPbBr_3_]_0.008_/Spiro‐OMeTAD/Au	1.190	82.8	25.80	25.40	40

**Figure 7:**
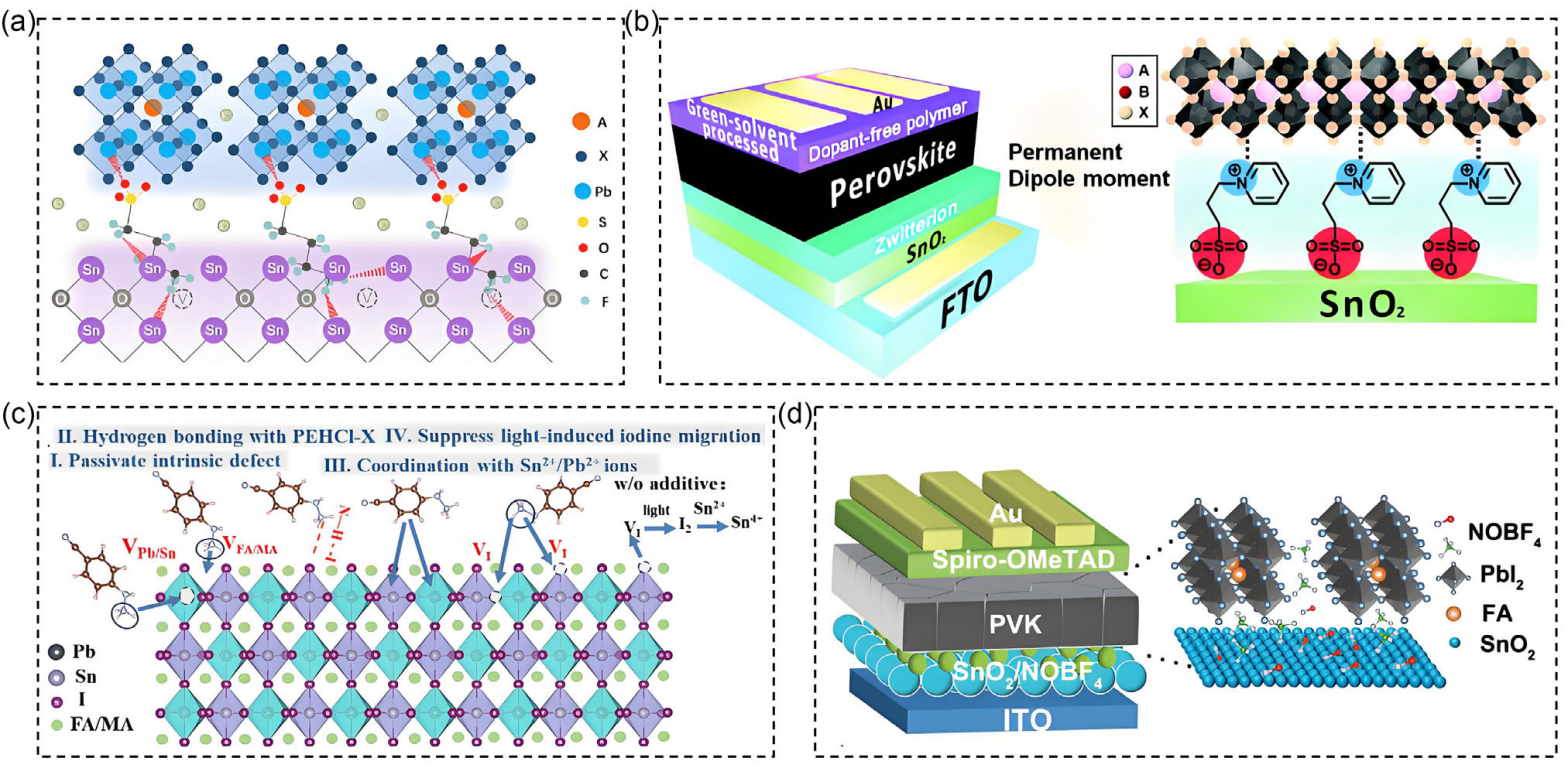
a) The possible interaction mechanism among KFBS, SnO_2_, and perovskite.^[^ ^93^ ^]^ Copyright (2022), with permission from Elsevier. b) Schematic illustration of the formation of a zwitterion on the SnO_2_ layer.^[^ ^94^ ^]^ Copyright (2018), with permission from Royal Society of Chemistry c) Schematic diagram of the passivation mechanism of PEHCl‐X.^[^ ^96^ ^]^ d) The schematic diagram of the device structure.^[^ ^97^ ^]^ Copyright (2023), with permission from Wiley‐VCH GmbH.

EWGs (such as –CF_3_, –CN) induce the downward shift of ETL conduction band through strong electronegativity, forming a more matching energy‐level difference with the perovskite conduction band and promoting efficient injection of photogenerated electrons.^[^ ^91^, ^92^ ^]^ Lan et al.^[^ ^93^ ^]^ incorporated multifunctional potassium nonafluoro‐1‐butanesulfonate (KFBS) molecules into the SnO_2_ ETL, improving energy level matching between the perovskite (PVK) layer and the ETL, while also passivating interface, grain boundary, and bulk defects (**Figure** **7**a). Park et al.^[^ ^94^ ^]^ used the amphoteric ionic compound 3‐(1‐pyridyl)‐1‐propane sulfonate to modify the SnO_2_ ETL, causing a work function shift in SnO_2_. The sulfonate group, with its strong electron‐withdrawing effect, effectively pulls electrons from the perovskite layer to the ETL/perovskite interface, enhancing electron transport (Figure 7b). Jung et al.^[^ ^95^ ^]^ found that NH_4_F could adjust the Fermi level of SnO_2_ and build a greater energy‐level gradient with perovskite layer.

In the SnO_2_ ETL, phenylhydrazine based additives containing electron‐withdrawing cyanide groups form strong coordination with Sn, stabilizing Sn—I bonds and passivating defects through electrostatic interactions with negatively charged vacancies. Time of flight secondary ion mass spectrometry showed that the iodine concentration in the ETL and silver electrodes treated with the modifier was significantly lower than that in the control group, indicating that PEHCl‐CN effectively prevented iodine diffusion (Figure 7c).^[^ ^96^ ^]^ Similarly, Zhang et al.^[^ ^97^ ^]^ introduced NOBF_4_ (tetrafluoroborate nitro) between the SnO_2_ and perovskite layers as an interfacial bonding layer, which passivated oxygen vacancies in SnO_2_ and significantly improved carrier extraction efficiency, achieving a high fill factor (FF) of 82.98% (Figure 7d). Luo et al.^[^ ^98^ ^]^ used multifunctional potassium trifluoromethanesulfonate (SK) to modify the buried interface between the chemical bath‐deposited (CBD) TiO_2_ and the perovskite layer. Their results demonstrated that SK, through the strong bonding interactions of the –CF_3_ and –SO_3_ groups, enhanced the adhesion between the perovskite and TiO_2_ ETL, acting as a crosslinker for the buried interface and improving the efficiency and stability of n‐i‐p PSCs. Bawendi et al.^[^ ^99^ ^]^ used volatile oxalic acid (OA) to replace the traditional thioglycolic acid (TGA) to enhance the attachment of SnO_2_ particles to the substrate and promote the subsequent condensation of colloidal particles. Fourier‐transform infrared tests showed that the volatile OA could be removed by thermal annealing and mild H_2_O_2_ treatment, effectively minimizing nonradiative carrier recombination at the interface and leading to a PCE of 24.6%. This strategy provides a novel approach for optimizing the CBD SnO_2_ layer to achieve high‐efficiency and stable PSCs.

For inverted PSCs, materials such as polystyrene, fullerenes, and their derivatives show excellent performance in modifying the ETL interface.^[^ ^100^ ^]^ Currently, the most popular ETL for the inverted PSCs is fullerenes (mainly C_60_) and their derivatives.^[^ ^101^, ^102^ ^]^ EWGs modification can not only improve the conductivity of fullerene derivatives, but also optimize the interface contact between ETL and perovskite.^[^ ^103^, ^104^ ^]^ In 2022, Zhan et al.^[^ ^105^ ^]^ designed a series of fullerene derivatives with terpene pyridine chelating groups, using expandable alkyl chains of different lengths as spacers FP Cn (*n* = 4, 8, 12) to replace PCBM as ETL. FP‐C8 exhibits the strongest molecular ordering and adhesion to perovskite surfaces, resulting in lower energy disorder and higher morphological stability compared to PCBM. Wei et al.^[^ ^104^ ^]^ designed a novel crosslinkable fullerene molecule, namely bis ((3‐methoxy‐3‐yl) methyl) malonate‐C_60_ monoadduct (BCM), as the ETL in PSCs. The intramolecular covalent interactions within CBCM films effectively prevent aggregation and enhance film density, exhibiting excellent electron mobility and a band structure suitable for efficient PSC. In 2024, Egelhaaf et al.^[^ ^106^ ^]^ used a novel fullerene based phosphonic acid (FAPA) as an interface dipole for electron selective contact and hole selective contact in PSC. This contact layer provides good charge selectivity and minimizes interface recombination of the bottom electrode.

In addition, hydrophobic EWGs can enhance the stability of fullerenes. Huang et al. demonstrated high waterproof performance of fullerene layers by bonding crosslinkable silane coupling agents with hydrophobic functional groups onto C_60_ substituted benzoic acid SAM (C_60_‐SAM).^[^ ^107^ ^]^ He et al.^[^ ^108^ ^]^ synthesized a cathode interface material with larger steric hindrance and strong electron‐withdrawing properties by linking phenanthroline with carbonlong (phenanthroline‐carbolong) units, which suppressed interface reactions with demethylated fullerene receptors. The D18 organic solar cells based on phenanthroline‐carbolong achieved a high efficiency of 18.2%, offering a new pathway for the fabrication of high‐efficiency, stable perovskite/organic tandem solar cells. The performance bottleneck of electron transport materials requires precise energy level control and interface defect passivation to fundamentally mitigate voltage loss and enhance damp heat stability. In the future, this approach will provide revolutionary interface engineering solutions for flexible devices and multijunction stacked cells.

## 4 Summary and Outlook

In summary, this article elaborates on recent research advancements in utilizing EWGs to enhance the optoelectronic performance of PSCs and analyzes the impact of EWGs on various interfacial layers within these devices. Broadly, the effects of EWGs materials on PSCs can be categorized into three aspects: 1) For the perovskite layer, EWGs enhance local electronegativity through electron‐withdrawing effects, induce interfacial dipole effects, and directly modulate electric field distribution to regulate defect passivation. 2) For the ETL, EWGs primarily optimize energy‐level gradients, improve charge extraction efficiency, and enhance electron transport. 3) For the HRL, electron‐withdrawing molecules prevent ion migration and aggregation via interfacial chemical bonding, thereby reducing nonradiative recombination. In addition, according to literature reports, the advantages and disadvantages of different types of EWGs are shown in the following **Table** **4**.

**Table 4:** Advantages and disadvantages of different types of EWGs.

EWG	Pros	Cons
‐CN	1) Strong binding of undercoordinated Pb^2+^ through N atoms with strong electronegativity; inducing the formation of a surface dipole layer and reducing the interface hole extraction potential barrier.	1) Decomposition leads to the generation of volatile cyanide compounds.
–SO_3_H	2) Dissociation releases protons, efficiently passivating iodine vacancies; multidentate chelation of Pb^2+^; strong hydrophilicity, promoting uniform film formation.	2) Unnecessary side reactions such as interface corrosion.
–C=O	3) Pb^2+^ coordination is mild and controllable; flexible molecular design; good solution compatibility.	3) The single point coordination strength is relatively weak.
Halogens	4) Easy to fill halogen vacancies; regulating bandgap; barrier against water and oxygen erosion.	4) Phase separation; evaporation at high temperatures.

**Figure 8:**
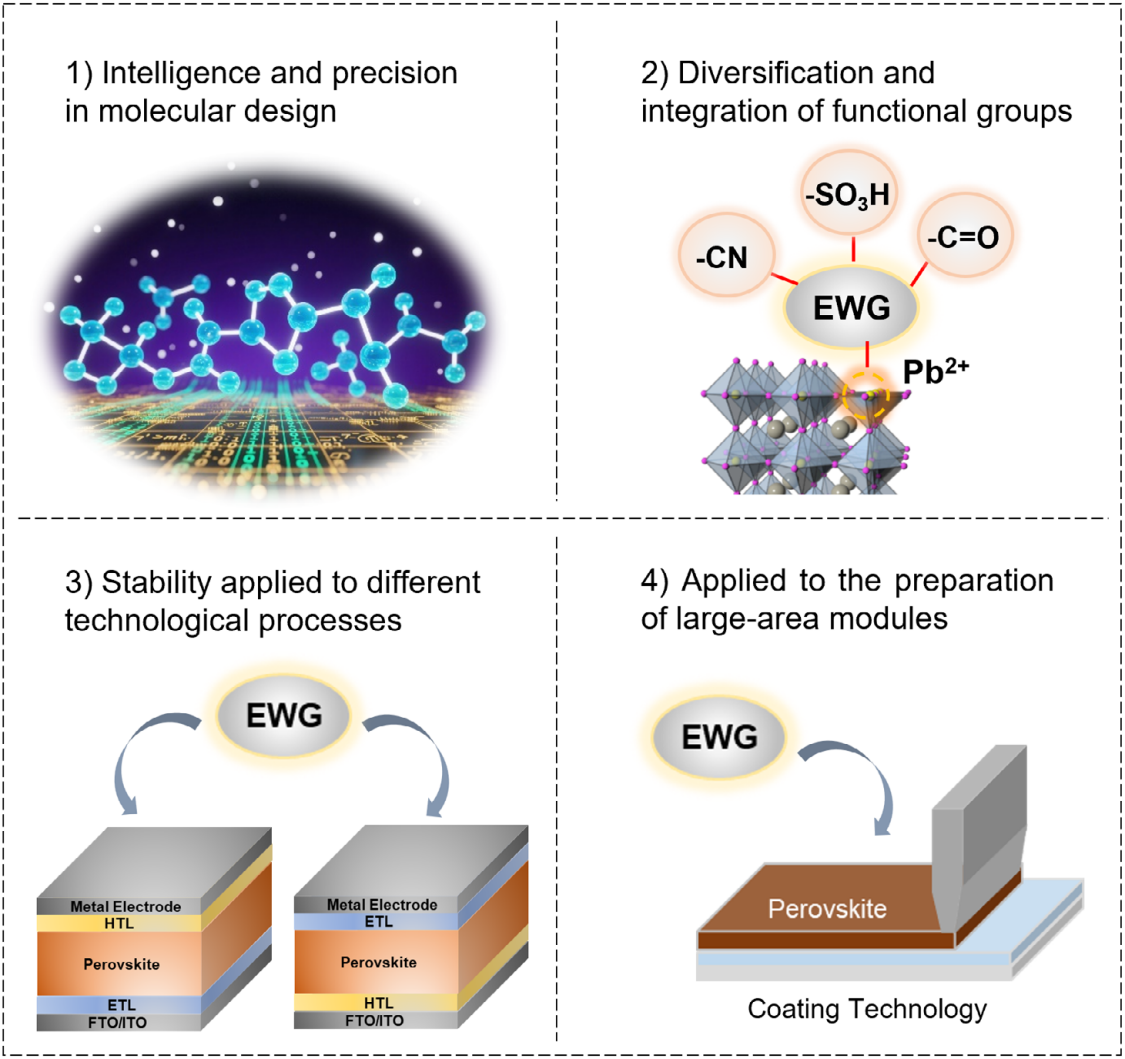
Summary of future prospects for EWMs.

In the field of PSCs, EWMs that containing fluorine, cyano, and/or sulfonic acid groups, demonstrate significant application potential due to their unique electronic modulation capabilities, particularly in boosting PCE and long‐term stability. However, despite their promise for performance optimization, industrial‐scale adoption still faces multiple challenges requiring interdisciplinary collaboration: 1) Insufficient correlation between molecular configuration and performance. The synergistic effects of electronegativity and steric hindrance (e.g., van der Waals volume of –CF_3_ ≈ 92 Å^3^)^[^ ^109^ ^]^ remain unquantified, hindering theoretical guidance for molecular design. 2) Conflict between process compatibility and crystallization kinetics. High additive concentrations of certain EWGs (e.g., > 5 wt%)^[^ ^15^ ^]^ disrupt perovskite crystallization, reducing grain size and inducing pinhole defects. 3) Environmental risks and scalability limitations. Some fluorine‐containing materials (e.g., PFOS derivatives) exhibit bioaccumulation risks (half‐life > 5 years),^[^ ^110^ ^]^ impeding industrial deployment. Ultimately, the deep integration of EWGs requires a holistic R&D framework encompassing “molecular design‐process optimization‐environmental assessment”: 1) Molecular design utilizes AI or machine learning for high‐throughput screening to ensure intelligent and accurate outcomes. 2) Investigating the diversification and integration of multiple electron‐withdrawing functional groups, with a focus on their synergistic effects. 3) Exploring the application of EWMs across diverse process systems to ensure universal and consistent modification effects. 4) Developing functional materials for perovskite large‐area module processes (e.g., coating, sputtering) (**Figure** **8**). With their ability to synergistically enhance efficiency, stability, and scalability, EWMs hold immense potential and broad prospects in advancing perovskite photovoltaics toward commercialization.

## Conflict of Interest

The authors declare no conflict of interest.
